# Machine Learning Approach for *Candida albicans* Fluconazole Resistance Detection Using Matrix-Assisted Laser Desorption/Ionization Time-of-Flight Mass Spectrometry

**DOI:** 10.3389/fmicb.2019.03000

**Published:** 2020-01-14

**Authors:** Margot Delavy, Lorenzo Cerutti, Antony Croxatto, Guy Prod’hom, Dominique Sanglard, Gilbert Greub, Alix T. Coste

**Affiliations:** ^1^Microbiology Institute, University Hospital Lausanne, Lausanne, Switzerland; ^2^SmartGene Services, EPFL Innovation Park, Lausanne, Switzerland

**Keywords:** machine learning, MALDI-TOF MS, *Candida albicans*, fluconazole resistance, diagnostic

## Abstract

*Candida albicans* causes life-threatening systemic infections in immunosuppressed patients. These infections are commonly treated with fluconazole, an antifungal agent targeting the ergosterol biosynthesis pathway. Current Antifungal Susceptibility Testing (AFST) methods are time-consuming and are often subjective. Moreover, they cannot reliably detect the tolerance phenomenon, a breeding ground for the resistance. An alternative to the classical AFST methods could use Matrix-Assisted Laser Desorption/Ionization Time-of-Flight (MALDI-TOF) Mass spectrometry (MS). This tool, already used in clinical microbiology for microbial species identification, has already offered promising results to detect antifungal resistance on non-azole tolerant yeasts. Here, we propose a machine-learning approach, adapted to MALDI-TOF MS data, to qualitatively detect fluconazole resistance in the azole tolerant species *C. albicans*. MALDI-TOF MS spectra were acquired from 33 *C. albicans* clinical strains isolated from 15 patients. Those strains were exposed for 3 h to 3 fluconazole concentrations (256, 16, 0 μg/mL) and with (5 μg/mL) or without cyclosporin A, an azole tolerance inhibitor, leading to six different experimental conditions. We then optimized a protein extraction protocol allowing the acquisition of high-quality spectra, which were further filtered through two quality controls. The first one consisted of discarding not identified spectra and the second one selected only the most similar spectra among replicates. Quality-controlled spectra were divided into six sets, following the sample preparation’s protocols. Each set was then processed through an R based script using pre-defined housekeeping peaks allowing peak spectra positioning. Finally, 32 machine-learning algorithms applied on the six sets of spectra were compared, leading to 192 different pipelines of analysis. We selected the most robust pipeline with the best accuracy. This LDA model applied to the samples prepared in presence of tolerance inhibitor but in absence of fluconazole reached a specificity of 88.89% and a sensitivity of 83.33%, leading to an overall accuracy of 85.71%. Overall, this work demonstrated that combining MALDI-TOF MS and machine-learning could represent an innovative mycology diagnostic tool.

## Introduction

*Candida albicans* is one of the most common opportunistic pathogens in humans (Naglik et al., 2011). Although *C. albicans* superficial infection are not life threatening, systemic infections can lead to a mortality up to 50% (Brown et al., 2012). In addition, antifungal resistance frequency among *C. albicans* is increasing worldwide (Pfaller et al., 2010; Castanheira et al., 2016). A recent study, based on data collected in the United States, concluded that even if it does not statistically improve patient outcome, an appropriate antifungal stewardship allows a significant reduction in antifungal use (Hart et al., 2019). Therefore, early detection of antifungal susceptibility is required to improve antifungal stewardship and to act against antifungal resistance rising. This is particularly pertinent regarding the recent emergence of the highly drugresistant *C. auris* (Spivak and Hanson, 2018; Kordalewska and Perlin, 2019).

Even if drug-resistance displays a lower incidence in fungi than in bacteria, it stays particularly worrying since the armamentarium against fungi is very limited since they, as eukaryotes, share quite a number of similar key biochemical characteristics. Thus, nowadays, only four antifungals classes are available: echinocandins, pyrimidine analog, polyenes and azoles. The first type of antifungal inhibits the cell wall biosynthesis, the second inhibits the fungal growth by nucleic acid destabilization and the two last disrupt the cell membrane integrity (Sanglard, 2016).

Azoles compounds are of particular concern since they are the first line treatment against non-life threatening *Candida* infections (Berkow and Lockhart, 2017). Indeed, although intrinsic resistance almost inexistent in *C. albicans*, acquired antifungal resistance can emerge, especially during long-term treatment (Cleveland et al., 2012; Sanguinetti et al., 2015).

Four main mechanisms of azole resistance have been described (Vandeputte et al., 2012; Berkow and Lockhart, 2017) relying on: (i) reduction of the fluconazole affinity with its target Erg11, due to mutation in its binding site (Sanglard et al., 1998); (ii) upregulation of *ERG11* expression, via a gain-of-function (GOF) mutation in the transcription factor (TF) Upc2, counteracting the fluconazole effects (Flowers et al., 2012); (iii) reduction of the drug concentration within the fungal cell by increased expression of multidrug transporters, thanks to GOF mutations in two TFs (Tac1 and/or Mrr1, respectively) (Coste et al., 2006; Dunkel et al., 2008); and (iv) alterations of the yeast metabolism (ex: a mutation in *ERG3)* (Martel et al., 2010). Development of antifungal drug resistance in *C. albicans* is a sequential process, via the acquisition of the different mutations along time, leading to highly resistant isolates (Coste et al., 2009). Resistance acquisition is probably favored by the phenomenon of tolerance (Delarze and Sanglard, 2015; Berkow and Lockhart, 2017). Indeed, azoles are fungistatic for *C. albicans*, which implies that this species is able to survive and to eventually grow at high fluconazole concentrations. As a consequence, residual growth (or trailing growth) can be observed at fluconazole concentrations higher than the minimum inhibitory concentration (MIC) (Delarze and Sanglard, 2015). Tolerance is dependent on the calcineurin pathway, as it can be inhibited using calcineurin inhibitor such as cyclosporine (Sanglard et al., 2003).

Although the genes involved in fluconazole resistance are well-known, the extensive diversity of the mutations that can occur makes difficult or even impossible to elaborate polymerase chain reaction (PCR)-based methods assessing *C. albicans* azole resistance (Morio et al., 2010; Ferrari et al., 2011; Vandeputte et al., 2012). Therefore, fluconazole resistance needs to be assessed *in vitro* by fastidious Antifungal Susceptibility Tests (AFST) based on the determination of the MIC, whose main disadvantage is its time to result of at least 24 h (Posteraro et al., 2013; Sanguinetti and Posteraro, 2014).

During the last 10 years, antifungal resistance detection by MALDI-TOF MS has been addressed (Marinach et al., 2009; De Carolis et al., 2012; Vella et al., 2013, 2017; Vatanshenassan et al., 2018). Marinach et al. (2009) have developed a method based on the changes occurring in *C. albicans* spectra after exposure to different concentrations of fluconazole. They determined a new alternative to the MIC, the minimal profile change concentration (MPCC). Based on the MPCC of several strains, new breakpoint concentrations (BPC) could be established, allowing the discrimination between resistant and susceptible strains. This assay was later modified and simplified, comparing only spectra obtained after 3 h of fungal exposure to three different antifungal concentrations: none, BPC, and high concentration (De Carolis et al., 2012; Vella et al., 2013, 2017). The Bruker company also developed recently a MALDI BioTyper Antibiotic Susceptibility Test Rapid Assay (MBT-ASTRA), that include antifungal resistance detection. MBT-ASTRA estimates the cellular growth of a sample after 6 h-exposure to an antifungal drug, based on the peaks’ intensities of the MALDI-TOF MS spectra compared to an internal standard as shown for *C. glabrata* (Sparbier et al., 2016; Vatanshenassan et al., 2018).

However, none of those studies clearly showed reliable results for detecting azole resistance in *C. albicans*. Indeed, for now, only a study has shown that MALDI-TOF MS was able to separate *C. glabrata* isolates according to their fluconazole susceptibility. However, this discrimination was based on a clustering of the spectra, and signature peaks have yet to be identified (Dhieb et al., 2015). Indeed, the main difficulty in assessing *C. albicans* azole susceptibility is the presence of a trailing effect due to the tolerance to azoles, which complicates AFST lecture.

Given these limitations, the aim of this study is to develop a MALDI-TOF MS procedure using machine learning to detect fluconazole resistance in *C. albicans* strains despite the tolerance phenomenon.

## Materials and Methods

### Sample Preparation

#### Strains

In this study, we used 12 pairs and 3 triplets of related *C. albicans* isolates (Table 1). The isolates from a given pair or triplet were isolated from the same patients while treated with antifungals. Related strains were documented by MultiLocus Sequence Typing (Sanglard et al., 1995; White et al., 1997; Coste et al., 2004, 2007, 2009; Dunkel et al., 2008; Posteraro et al., 2009; Lohberger et al., 2014). The fluconazole susceptibility status was defined using the EUCAST breakpoints using thebroth microdilution method (Sanglard et al., 1995; White et al., 1997; Coste et al., 2004, 2007, 2009; Dunkel et al., 2008; Posteraro et al., 2009; Lohberger et al., 2014; EUCAST, 2018).

**TABLE 1:** *Candida albicans* strains used in the project.

**Patient**	**Set**	**ID**	**MIC FLC**	***CDR1/CDR2***	***MDR1***	***TAC1***	***ERG11***	***MRR1***	***UPC2***	***Source***
1	TT	2321	0.25				^∗^			Coste et al., 2007
	TT	2322	16	X		X	X			
	V	2323	32	X		X	X		X	
2	TT	731	0.25							Coste et al., 2007
	TT	732	16	X		X				
	V	735	64	X		X				
6	TT	2243	1				X			Coste et al., 2009
	V	2242	8	X		X	X			
10	TT	741	0.25							Posteraro et al., 2009
	V	742	16		X			X		
12	V	2284	0.25							Dunkel et al., 2008
	TT	2285	16	X	X	X		X		
19	TT	290	0.5							Sanglard et al., 1995
	TT	292	128		X		X	^∗^		
20	V	294	0.25							Coste et al., 2004
	TT	296	128	X		X	X			
21	V	347	0.25							Coste et al., 2009
	TT	288	0.5				X			Sanglard et al., 1995
	TT	289	128	X		X	X			
22	TT	3534	0.5							White et al., 1997
	TT	3548	128	X	X	X	X	X		
4	V	750	16		X			^∗^		Posteraro et al., 2009
	TT	2250	1				X			
5	V	757	2			^∗^				Coste et al., 2009
	TT	758	16	X		X	^∗^			
9	TT	482	8				^∗^			Coste et al., 2009
	TT	488	16	X		X	X			
13	TT	520	32	X		X				Coste et al., 2009
	TT	522	128	X		X	X			
15	TT	2250	1				X			Coste et al., 2009
	TT	2251	16	X		X	X			
18	TT	281	1				^∗^			Coste et al., 2009
	TT	284	32	X		X	X			

*The strains with the same patient number were isolated from the same patient along fluconazole treatment time. The fluconazole (FLC) Minimum Inhibitory Concentration (MIC) is given in μg/mL. The fluconazole-susceptible strains are highlighted by a light green background and the resistant strains by a white one. A cross represents a homozygous mutation whereas a star represents a heterozygous mutation. All the strains are DSY strains. TT means that the strain was part of the training or the testing set of the final model and the V means that the strain was part of the validation set of the final model.*

#### Sample Preparation

The sample preparation procedure and the protein extraction protocol are described in Supplementary Data Sheet S1 – Sample preparation. All samples were prepared at least in pair of biological replicates.

### MALDI-TOF MS Analysis

#### MALDI-TOF MS Settings

The MALDI-TOF MS spectra were acquired on a Bruker Daltonic Microflex LT mass spectrometer device in technical duplicates, as described in Supplementary Data Sheet S1 – MALDI-TOF MS settings. MALDI-TOF MS settings are presented in Supplementary Table S1. Therefore, for each strain, we obtained spectra from two biological replicate, each in technical duplicate.

#### Quality Controls

A first quality control was based on the *C. albicans* identification log(scores)obtained with the MALDI Biotyper Compass explorer software (v.4.1, Bruker). The raw spectra were imported in the software and automatically compared to all the spectra available in the database 2017. Only the spectra with a logarithmic score [log(score)] equal or higher than 1.70 for *C. albicans* were conserved for the following steps (for review, see Bader, 2017).

A second quality control was performed using CCI matrices (QC2) generated with the CCI tool of the MALDI Biotyper Compass explorer software. It uses a cross-correlation method to analyze the relationship between different spectra (Arnold and Reilly, 1998). It was performed on each pair or triplet of clinical strains treated in the same conditions. First, the variability between the replicates was evaluated by calculating the CCI scores obtained between the spectra of each technical duplicate. If the CCI score was below 0.75, the spectra of the corresponding technical duplicate were removed. Secondly, the biological variability was assessed by calculating the mean of CCI scores obtained between biological replicates. If the mean of CCI scores was below 0.5, the replicate was removed. It has to be noted that the thresholds were arbitrary set following our preliminary observation (data not shown). The parameters of the CCI tool are the same than in De Carolis et al. (2012).

#### R Version

The spectra processing and analyses were performed in the R environment (v. 3.6.1) with R studio (v. 1.1.453) (RStudio Team, 2016; R Core Team, 2019).

### Spectra Processing

#### Housekeeping Peaks

A list of peaks, called *C. albicans* “housekeeping peaks” because they are presents in all the spectra originated from *C. albicans*, were used for the spectra processing. To obtain them, we extracted the peaks list of the 30 *C. albicans*’ superspectra from the Bruker 8 database (2019). Then, peaks present in at least 70% of the superspectra by using a tolerance of position of ± 3 m/z were assessed as *C. albicans* housekeeping peaks (Table 2).

**TABLE 2:** Housekeeping peaks and their associated frequency in the Bruker *C. albicans* superspectra.

**Hkpeak**	**Frequency**
**3489.66**	0.852
**3511.72**	0.818
**4381.44**	0.7912
**6060.56**	0.915
**6111.43**	0.829
**6364.35**	0.812
**6465.88**	0.837
**6617.99**	0.969
**6906**	0.7064
**6980.82**	0.967
**7022.92**	0.882
**8761.14**	0.812

#### Spectra Processing

The spectra were treated with an R script based on the MALDIquant package [version (v.) 1.18] created by Gibb and Strimmer (2012). The spectra were imported in R with the MALDIquantForeign package (v. 0.11.5; Gibb and Franceschi, 2018) and treated separately, depending on the condition in which they were acquired (MAX-CYCLO, BPC-CYCLO, NULL-CYCLO, MAX-NoCYCLO, BPC-NoCYCLO, and NULL-NoCYCLO). The peaks intensities were exported under the form of a different intensity matrix for each condition, which contains the intensities for all the peaks in each spectrum. The description of the spectra processing is described Figure 1 in Supplementary Data Sheet S1 – Spectra processing.

**FIGURE 1:**
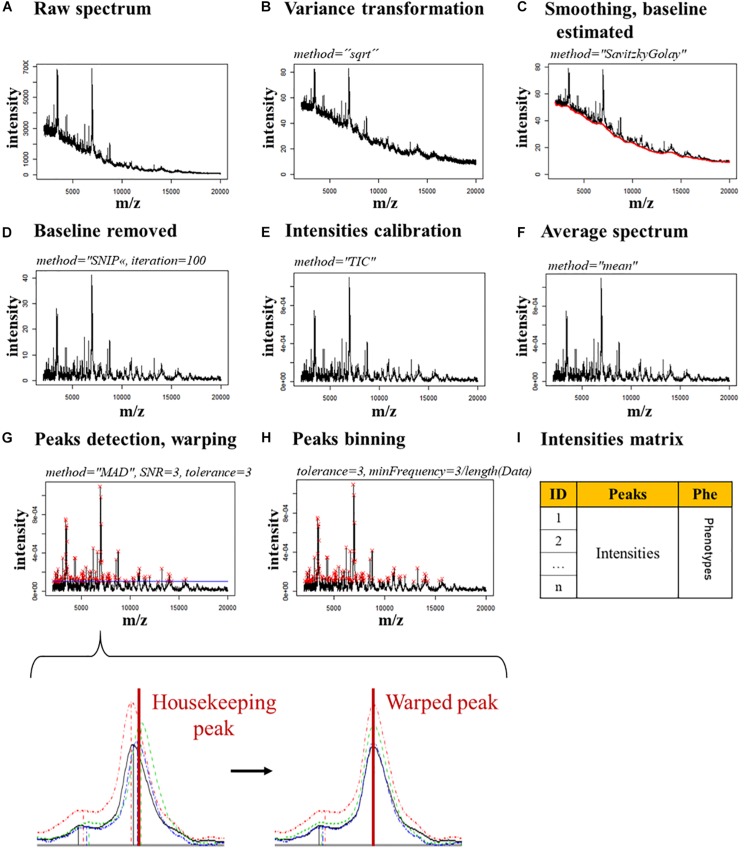
Spectra processing pipeline. The parameters used for each step are indicated in italics. **(A)** Raw spectrum. **(B)** Raw spectrum’s variance is transformed. **(C)** The spectrum is smoothed and the baseline (red line) is estimated. **(D)** The baseline is removed. **(E)** The spectrum’s intensities are calibrated. **(F)** The spectra of the technical replicates are merged in a single average spectrum. **(G)** The peaks (red crosses) are detected and warped on the housekeeping peaks, which allow a stable alignment. A zoom of a single peak shows the changes expected in the alignment of a housekeeping peak after the warping. **(H)** The peaks are binned by merging together the peaks closer than 3 m/z. **(I)** An intensity matrix is generated with the intensities of each peak, for each spectrum.

### Machine-Learning Approach

#### Data Preparation

For each condition (MAX-CYCLO, BPC-CYCLO, NULL-CYCLO, MAX-NoCYCLO, BPC-NoCYCLO, and NULL-NoCYCLO), the intensity matrix was randomly split by strains in three data sets: a training set, containing spectra corresponding to 50% of the strains, a testing set (25%) and a validation set (25%). To ensure than the number of fluconazole resistant and fluconazole susceptible strains were balanced in each set, the ratio of the number of fluconazole resistant strains versus the number of susceptible strains was forced between 0.667 and 1.5.

#### Peaks Selection

The peaks were ranked by their associated Mean Decrease in Gini index obtained after performing a Random Forest (RF) classifier with the *randomForest* function (randomForest package v. 4.6-14, Cutler et al., 2018) on the training set. Three values of number of trees to grow (ntree) were tested (500, 1000, and 2000). The other default parameters of the *randomForest* function were conserved. Four subsets of peaks were selected for each condition and each ntree value, depending of their rank: all the peaks (Mean Decrease in Gini index equal of above 0, iThr = 0) and peaks associated to a Mean Decrease in Gini index equal of above 0.3, 0.4, and 0.5, respectively (iThr = 0.3, iThr = 0.4, and iThr = 0.5).

#### Models Testing

For each condition (MAX-CYCLO, BPC-CYCLO, NULL-CYCLO, MAX-NoCYCLO, BPC-NoCYCLO, and NULL-NoCYCLO), the prediction accuracy of the RF classifier (*randomForest* function, randomForest package v. 4.6-14, Cutler et al., 2018), the logistic regression (*glm* function, R v. 3.6.1.) and the Linear Discriminant Analysis (LDA, *lda* function, *MASS* package v. 7.3-51.4,Ripley et al., 2019) was tested. Each method was performed on the subsets of peaks created in 2.5.2 (iThr = 0, 0.3, 0.4, and 0.5), leading to a total of 32 models by condition, meaning 192 pipelines of analysis from sample preparation to resistance prediction. The prediction accuracy of each pipeline was stored. For RF, the default parameters of the *randomForest* function were conserved except for the ntree where three values were tested (500, 1000, and 2000). For the logistic regression, the family parameter of the *glm* function was set on “binomial” and the other default parameters were conserved. Finally, for the LDA, the default parameters of the *lda* function were conserved.

#### Selection of the Most Accurate Pipelines

Once all the above described pipelines were generated, the 15% models with the highest accuracies were selected. If the machine-learning models differed only by the ntree or the iThr parameter applied, only the pipeline associated to the best accuracy was conserved for the following step.

#### Assessment of the Models’ Robustness

To test the robustness of the 15% most accurate pipelines, the training and testing set of the intensity matrix associated to each pipeline were merged and the strains associated were randomly split (ratio 2:1) in new training and testing sets. Balancing of the data was ensured as in 2.5.1.

The corresponding model was then trained on the new training set and the accuracy of the susceptibility level prediction on the testing set was stored. This process was iteratively repeated 100 times.

The pipeline associated with the highest accuracy and the lowest variance accuracy was extracted and trained on the training and testing set merged together. The pipeline’s parameters were stored for validation.

#### Pipeline Validation

The final pipeline of analysis was applied to predict the fluconazole susceptibility level on the validation set [*predict* function, stats package v. 3.6.1 (R Core Team, 2019)]. The predictions were challenged with the known fluconazole susceptibility levels of the strains and the accuracy, specificity, and sensitivity were calculated.

#### Data Storage

Intensity matrices datasets are available on FigShare (doi: 10.6084/m9.figshare.9900896).

Script and final model can be found on GitHub^1^.

## Results

### Optimization of Sample Preparation and Spectra Acquisition

The first step of the analysis is the acquisition of good quality spectra, evaluated by the accuracy of *C. albicans* identification [identification log(scores)]. This constitute the first quality control (QC1- Supplementary Figure S1A). In this end, the protocol implemented by De Carolis et al. (2012), using a formic acid (FA)-based protein extraction, was compared to a protocol using a mechanical glass bead-based extraction, on a subset of two related strains (DSY290/DSY292). In each case, different volumes of fungal suspension (FS) and FA were tested.

Independently of the FS and FA volumes used, the bead-based extraction allowed the acquisition of better-quality spectra (Welch two sample *t*-test: *p*-value = 3.0 × 10^–11^), with 87.10% of the spectra being correctly identified as belonging to *C. albicans*, against only 49.62% for the FA-based extraction (Supplementary Figure S2A). Between the spectra obtained with the bead-based extraction protocols, better log(scores) were obtained for the ones treated with the 10 μl of FA (Two-Way Crossed ANOVA, *p*-value = 10.0 × 10^–10^), with 94.44% of accurate identification against 76.92% for the bead-based extractions using only 2 μL of FA (Supplementary Figure S2B). This shows the importance of thoroughly break the yeast cell wall by mechanical extraction in order to obtain a higher number of mass profiles, in contrast to bacteria.

As a final protocol option, we chose the bead-based extraction protocol using 10 μL of FA and 0.5 mL of FS. Indeed, although there were no significant differences of log(scores) with the bead-based protocol using 10 μL of FA and 1 mL of FS, all the spectra acquired after using the chosen protocol were correctly identify as belonging to *C. albicans*, whereas only 88.89% of the spectra were correctly identified with the protocol using 1 mL of FS (Supplementary Figure S2C).

The second step, also called quality control 2 (QC2 – Supplementary Figure S1B) was to ensure the spectra’s technical and biological reproducibility. It was performed using CCI matrices, generated for each pair or triplet of clinical strains treated in the same conditions.

The systematic application of these two QCs (Supplementary Figure S1) will ensure that the spectra are of similar quality and can be compared.

The main pitfall of the fluconazole resistance measurement for *C. albicans* is the trailing phenomenon due to fluconazole tolerance. In this regard, we compared samples exposed to cyclosporin A (CYCLO, 5 μg/mL), a calcineurin inhibitor, to untreated samples (NoCYCLO). In each case, three fluconazole concentrations were tested: a maximum concentration (MAX, 256 μg/mL), which was superior to the maximal concentration usually used to determine the MIC, a null concentration (NULL, 0 μg/mL) and a breakpoint concentration, known to allow discrimination of susceptible and resistant strains spectra by CCI matrix (BPC, 16 μg/mL, Elena De Carolis, *personal communication*, De Carolis et al., 2012). This led to six final conditions: MAX-CYCLO, BPC-CYCLO, NULL-CYCLO, MAX-NoCYCLO, BPC-NoCYCLO, and NULL-NoCYCLO.

Using optimized protocol, we acquired 1366 spectra with at least two biological replicates for each strain passing both QCs. First, 1363 out of 1366 (97,2%) passed QC1, showing the efficiency of the glass beads sample’s preparation protocol to acquire high quality spectra. Then only 953 spectra out of 1363 passed QC2 (69,2%) with 422 acquired from fluconazole resistant strains and 431 from fluconazole susceptible strains.

### MALDI-TOF MS Database Implementation

To assign and quantify peaks for each MALDI-TOF MS spectra, R scripts were developed based on the MALDIquant package created by Gibb and Strimmer (2012). The 953 quality-controlled spectra were separated by condition (77 for MAX-CYCLO, 82 for BPC-CYCLO, 83 for NULL-CYCLO, 127 for MAX-NoCYCLO, 92 for BPC-NoCYCLO and 97 for NULL-NoCYCLO) and processed as described in Section “Machine-Learning Approach.”

We thus obtained a database constituted of six subsets or six intensity matrices (one by fluconazole and cyclosporin condition). Each subset contained the filename, the strain and the fluconazole susceptibility level (resistant or susceptible) of the analyzed samples, in additions of the intensities of each peak. This corresponded to 364 peaks for 82 average spectra for BPC-CYCLO, 336 peaks for 77 average spectra for MAX-CYCLO, 354 peaks for 84 average spectra for NULL-CYCLO, 369 peaks for 92 average spectra for BPC-NoCYCLO, 404 peaks for 127 average spectra for MAX-NoCYCLO, and 382 peaks for 97 average spectra for NULL-NoCYCLO.

### Fluconazole Resistance Detection by Machine-Learning Approach

**FIGURE 2:**
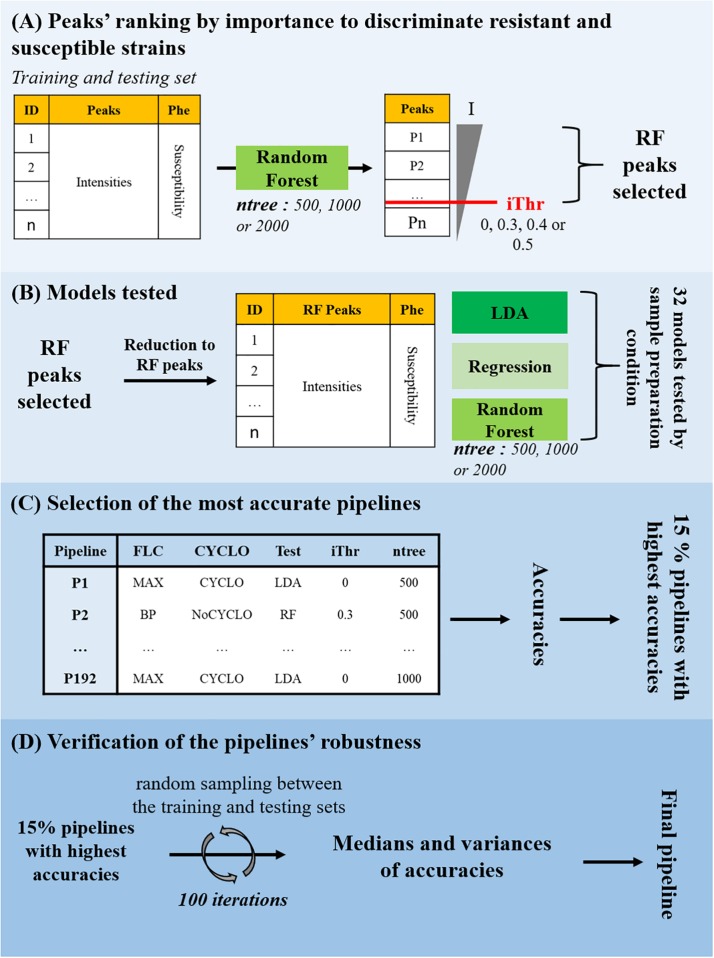
Fluconazole resistance detection by machine-learning approach. **(A)** Peaks’ ranking by importance to discriminate resistant and susceptible strains. A model based on the Random Forest (RF) classifier was trained on the training set and tested on the testing set to separate the fluconazole-resistant strains from the fluconazole-susceptible ones depending on the peaks’ intensities. Three values of number of trees to grow (ntree) were tested. The peaks were ranked by their associated Mean Decrease in Gini index (I) and four Decrease in Gini index thresholds (iThr = 0, 0.3, 0.4, 0.5) were arbitrarily set to extract a list of discriminating peaks (RF Peaks). **(B)** Models testing. The intensity matrix was reduced to the RF peaks and RF, logistic regression and LDA models were trained and tested to separate the fluconazole-resistant strains from the fluconazole-susceptible ones depending on the peaks’ intensities. In total, 32 models were tested on each of the 6 subsets, for a total of 192 pipelines of analysis from sample preparation to resistance prediction, each associated to a specific accuracy. **(C)** Selection of the most accurate pipelines. The 15% pipelines corresponding to the highest accuracies were selected. **(D)** Verification of the pipelines’ robustness. The training and testing set associated to each of the 15% best accurate pipelines were merged and randomly split (ratio 2:1) in new training and testing sets. The model was trained on the new training set and the accuracy of the susceptibility level prediction on the testing set was stored. This process was iteratively repeated 100 times to generated as many different training/testing set combinations. The pipeline associated with a high median of accuracies and a low variance of accuracies was selected for validation.

In order to determine which machine-learning approach would be more appropriate to detect fluconazole resistance on MALDI-TOF MS spectra, we compared three algorithms: RF, logistic regression and LDA. These algorithms were either tested onon complete intensity matrices or on 3 reduced ones, each containing a selection of important peaks. These relevant peaks were selected by a first RF round (see Sections “Data Preparation,” “Peaks Selection,” and “Models Testing” Figure 2). This led to 32 models to be tested on the 6 subsets (MAX-CYCLO, BPC-CYCLO, NULL-CYCLO, MAX-NoCYCLO, BPC-NoCYCLO, and NULL-NoCYCLO), leading to 192 pipelines of analysis from sample preparation to spectra analysis.

**FIGURE 3:**
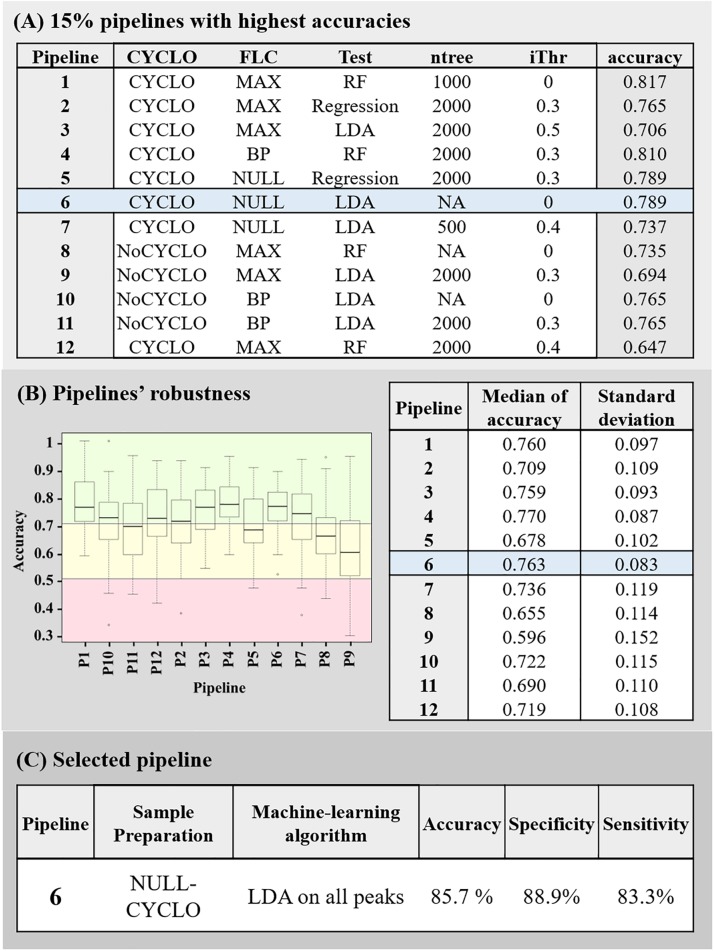
Summary of the pipelines selected with the machine-learning approach. **(A)** 15% pipelines with the highest accuracy. Each line of the table described the sample’s preparation conditions (Cyclo and FLC), the algorithm (Test), the Mean Decrease in Gini index threshold (iThr) and number of trees (ntree) parameters used in the pipeline and the accuracy associated to it. **(B)** Pipelines’ robustness. Graph of the accuracies obtained by each 15% best pipelines during the 100 rounds they were submitted to, and summary of the associated median and variances of accuracies. The red box represents accuracy below 50%, the yellow box, the accuracies between 50 and 70% and the green box, the accuracies above 70%. **(C)** Description of final selected pipeline’s parameter and its associated accuracy, specificity, and sensitivity.

Then, the accuracies of all the 192 pipelines tested, were compared and the 15% pipelines associated to the highest accuracies were selected (Figure 2C). If pipelines differed only be the ntree or the iThr parameter applied, only the model associated to the best accuracy was conserved for the following step. At this point, 12 pipelines were selected. As illustrated in Figure 3, most of the selected pipelines correspond to samples treated with cyclosporin.

Next, the robustness of these 12 pipelines were tested (see Section “Assessment of the Models’ Robustness”). At this point, the pipeline 4 (CYCLO-BP, RF, ntree = 2000 and iThr = 0.3) and 6 (CYCLO-NULL, LDA, iThr = 0) presented similar success of prediction. Pipeline 4 displayed an accuracy of 77.0 ± 8.7% and pipeline 6 an accuracy of 76.3 ± 8.3%. We selected the pipeline 6 as all the peaks of the spectra were considered by the LDA analysis.

Finally, the pipeline 6 parameters were extracted after training on all the strains spectra, except the initially excluded validation set one, in order to compensate the relative few numbers of spectra available and thus increase the robustness of the model.

### Validation

To validate the pipeline 6, we imported the initially determined validation set to predict the fluconazole susceptibility of each replicate. Ten out of 12 fluconazole resistant replicates and 8 out of 9 fluconazole-susceptible replicates were correctly categorized, leading to an overall accuracy of 85.71%, a specificity of 88.89% and a sensitivity of 83.33%.

## Discussion

During this project, we first optimized a protein extraction protocol that allowed the acquisition of MALDI-TOF MS high quality spectra and implemented two quality controls to assess the spectra quality. In a second part of the project, we conceived an R pipeline based on Gibb’s work (Gibb and Strimmer, 2012) to treat the spectra acquired with MALDI-TOF MS and to allow their comparison. Finally, we compared the accuracy of 192 pipelines of analysis, using machine-learning algorithms, to detect fluconazole resistance in *C. albicans*. We ended up with a final pipeline, which could be completed in less than 4 h. Samples were treated for 3 h with cyclosporin A in absence of fluconazole and acquired spectra were analyzed by an LDA algorithm on all the peaks. This model reached a specificity of 88.89% and a sensitivity of 83.33%, leading to an overall accuracy of 85.71%.

Surprisingly, the selected model is based on cyclosporin A treatment alone. On one hand, cyclosporin A treatment is crucial as reduction of the incubation time led to spectra which were systematically assigned as resistant by our model (data not shown). Therefore, a minimal incubation time, probably affecting proteins synthesis, is required to discriminate resistant from susceptible isolates. This confirms that cyclosporin A, that acts through the calcineurin pathway, induces differential protein expression between susceptible and resistant strains. Previous studies effectively shown that calcineurin is involved in tolerance but also resistance through the Pkc1 and TOR pathways (Cowen et al., 2006; LaFayette et al., 2010; Robbins et al., 2010). Transcriptional profiling data performed either on strains in the presence or absence of calcineurin or carried out on azole-susceptible or -resistant isolates, highlighted the differential expression of heat-shock or ribosomal encoding genes (Coste et al., 2004; Karababa et al., 2004, 2006). These gene products are the main proteins detected by MALDI-TOF MS analysis (Croxatto et al., 2012). On the other hand, fluconazole treatment appeared to be unnecessary. Hoehamer et al. (2009) previously showed that azole susceptible and resistant isolates could be differentiated by protein expressions in absence of any treatment. Those discriminating proteins were identified by 2D gels electrophoresis and MALDI-TOF MS analysis. Hoehamer et al. (2009) also showed that the discriminating proteins were specific to the underlying azole resistance mechanisms. However, they used a higher range of m/z than used in routine and in this study (Hoehamer et al., 2009). Nevertheless, we also attempted to discriminate strains based on their underlying resistance mechanisms but without success (data not shown). Indeed, the limited numbers of isolates available for each mechanism impaired the machine-learning analysis, which required high number of samples to be efficiently trained.

In this regard, our approach presents two main pitfalls. The first is the efficiency of the peaks positioning. This positioning relies on the warping. The 12 housekeeping peaks used to warp the spectra are a relatively low number to ensure a stable peaks positioning. This step is however crucial since it guarantees a satisfactory recovery of the peaks then used by the LDA algorithm. To assess the efficiency of this warping step, 6 averaged spectra acquired independently of the ones used to build the pipeline were subsequently aligned with the database spectra and processed. On all of them, the peaks positioning was efficient and 5 out of 6 were efficiently classified despite not having been trimmed by QC2 (data not shown). Indeed, by allowing a peak position’s tolerance of 3 m/z, we overcame the small spectra variations.

Second, we acquired spectra from a relatively small number of strains, which is not optimal for a machine-learning training step. To get around this issue, the robustness step was introduced to further validate the machine-learning models. Ideally, the databases should be enriched to further train the selected LDA model. This is indeed the main principle of machine-learning approaches (Jordan and Mitchell, 2015). One step further, this increase of database size with well-characterize strains would allow to train models to discriminate between the different possible azole resistance mechanisms, since, as mentioned before, spectra changed upon their occurrence (Hoehamer et al., 2009).

Altogether, this study acts as a proof-of-principle in the mycology field. This machine-learning approach could be applied to predict resistance from MALDI-TOF MS data on other fungi- antifungals associations. This offers a new qualitative diagnostic tool with same-day results delay. This allows a better patient care and a reduced amount of antifungal MIC determination, focusing only on the few predicted resistant strains.

## Data Availability Statement

Intensity matrices datasets are available on FigShare (doi: 10.6084/m9.figshare.9900896). Script and final model can be found on GitHub (https://github.com/mDelavy/MALDIresistance-PAPER).

## Author Contributions

ATC, MD, GG, LC, AC, and DS contributed to the conception and design of the study. MD organized the database. MD and LC performed the statistical analysis. ATC and MD wrote the first draft of the manuscript. GG, GP, and LC wrote sections of the manuscript. All authors contributed to the manuscript revision, read and approved the submitted version.

## Conflict of Interest

LC was employed by the company SmartGene. The remaining authors declare that the research was conducted in the absence of any commercial or financial relationships that could be construed as a potential conflict of interest.

## Footnotes

1 https://github.com/mDelavy/MALDIresistance-PAPER
