# Supplementary material for: Machine Learning Approach for Candida albicans Fluconazole Resistance Detection Using Matrix-Assisted Laser Desorption/Ionization Time-of-Flight Mass Spectrometry
Source: Front Microbiol. 2020 Jan 14;10:3000. doi: 10.3389/fmicb.2019.03000 (PMC6971193; doi:10.3389/fmicb.2019.03000)
Supplement: Supplementary file 1 [file Data_Sheet_1.docx]

Supplementary Material – S1

# Sample preparation

Fluconazole solutions at 32μg/mL and 512μg/mL were prepared in Rosewell Park Memorial Institute medium (RPMI-1640, Sigma-Aldrich, Saint-Louis, MO, USA), buffered with 0.165 mol/L of 3-(*N-*morpholino) propanesulfonic (MOPS) acid and complemented with 2% glucose (Sigma-Aldrich), diluted 2 times (RPMI 1x), from a fluconazole stock solution in water at 2.56 mg/mL. Cyclosporin A solution at 10mg/mL in dimethyl sulfoxide (DMSO, (CH3)2SO PanReac AppliChem, Darmstadt, Germany) was prepared from a dry material (Cyclosporin A, *Tolypocladium inflatum*, Sigma-Aldrich, Saint-Louis, MO, USA).

Liquid cultures (overnight incubation in 3mL of Yeast Extract Peptone Dextrose (YEPD), 30°C, 200rpm) were centrifuged at 4600 rpm for 5 min and washed twice in 3 mL of Phosphate-buffered saline (PBS, Bichsel, Interlaken, Switzerland). Yeast cells pellet was resuspended in 3 mL RPMI 1x. Then, 500 or 1000 μL of the yeast suspension (McFarland = 2.0) was mixed with either the same volume of RPMI 1x (NULL) or two fluconazole solutions (MAX: 512μg/mL, BPC: 32μg/mL) for a final concentration of MAX: 256μg fluconazole/mL or BPC: 16 μg fluconazole/mL. Finally, 0.5 μL of water (NoCYCLO) or cyclosporin A solution (CYCLO) at 10 mg/mL was added to obtain a final concentration of 5μg/mL. The samples were incubated for 3 h at 37°C under shaking (300 rpm). After incubation, samples were centrifuged at 14’000 rpm for 5 min at 4°C and washed twice in 1 mL H_2_O. After the last centrifugation pellet was resuspended in the remaining H_2_O and 2 or 10μL of 70% formic acid (FA). In addition of the FA extraction, a bead-based extraction method was also tested. In this case, acetonitrile was added in the same volume than FA. For each sample, 0.1 g of acid-washed glass beads (Sigma-Aldrich) were added. The cells were lysed by shaking in a Precellys^®^ Evolution homogenizer (Bertin instruments, Montigny-le-Bretonneux, France) at 6500 rpm, 1x30s, 1x60s and 1x90s, with 30s rest between each round. The samples were then centrifuged at 14’000 rpm for 5 min at 4°C and the supernatant was used for MALDI-TOF MS spectra acquisition.

# MALDI-TOF MS settings

The MALDI-TOF MS spectra were acquired on a Bruker Daltonic Microflex LT mass spectrometer device. One μL of the protein extracts was loaded on a 96 wells MALDI target plate (MFX μFocus MALDI plate 96 circles 2400μm NA07, HIST Inc.) and air dried.  Then, for the MALDI-TOF detection, 1μL of α-Cyano-4-hydroxycinnamic acid matrix (HCCA portioned, Bruker Daltonik, 2.5 mg), resuspend in 250μL of standard solvent (acetronitrile 50%, H_2_O 47.5% and trifluoracetic acid 2.5%, Sigma-Aldrich) was deposited on each well to cover the protein extracts. The plate was left in ambient conditions until the matrix solution was completely dry. The plate was then loaded in the microflex device and was ready to be analyzed. The spectra were acquired using the FlexControl software. The settings used are described in Supplementary Table 1 (Elena De Carolis, *personal communication*). In optimal conditions, 600 laser shots were acquired per well to compute a spectrum. The spectra were exported as mzXML files with the FlexAnalysis software (version (v.) 3.4, build 76, Bruker) for further R analysis. Each sample was analyzed in technical duplicate.

# Spectra processing

The spectra were treated with an R script based on the MALDIquant package (version (v.) 1.18) created by Gibb *et al.* (Gibb and Strimmer 2012). The spectra were imported in R with the MALDIquantForeign package (v. 0.11.5, (Gibb and Franceschi 2018) and treated separately, depending on the condition in which they were acquired (MAX-CYCLO, BPC-CYCLO, NULL-CYCLO, MAX-NoCYCLO, BPC-NoCYCLO and NULL-NoCYCLO).

The intensities’ variance was transformed (*transformIntensity* function*,* method = “sqrt”), the spectra were smoothed (*smoothIntensity* function*,* method = “SavitzkyGolay”, halfWindowSize = 10 m/z) and the baseline was removed (*removeBaseline* function, method = “SNIP”, iteration = 100). The intensities were calibrated (*calibrateIntensity* function*,* method = “TIC”) and the technical duplicates were merged in a single average spectrum (*averageMassSpectra* function*,* method = “mean”). The peaks were detected (*detectPeaks* function*,* method = “MAD”, halfWindowSize = 10 m/z, SNR = 3), warped on the housekeeping peaks (*determineWarpingFunctions, warpMassPeaks* and *warpMassSpectra* functions, warpingTolerance = 3m/z) and binned (*binPeaks* function, tolerance = 3 m/z). The peaks intensities were exported under the form of an intensity matrix (i*ntensityMatrix* function), which contains the intensities for all the peaks in each spectrum. The matrix of intensity thus obtained was normalized by the median intensity of each spectra to reduce variability between samples, as shown before (Padoan et al. 2018).
