# Supplementary material for: Machine Learning Approach for Candida albicans Fluconazole Resistance Detection Using Matrix-Assisted Laser Desorption/Ionization Time-of-Flight Mass Spectrometry
Source: Front Microbiol. 2020 Jan 14;10:3000. doi: 10.3389/fmicb.2019.03000 (PMC6971193; doi:10.3389/fmicb.2019.03000)
Supplement: Supplementary file 2 [file Image_1.pdf]

**(A)**

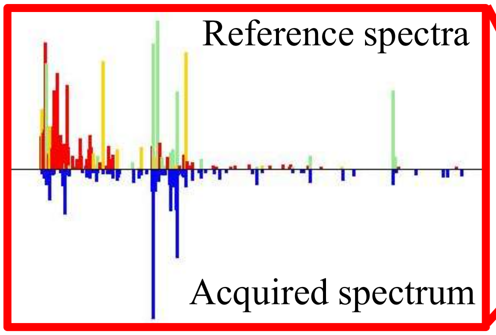


**DSY292 – No CYCLO**

| MAX | | BPC | | NULL | |
|---|---|---|---|---|---|
| 2.04 | 1.96 | 1.90 | 1.88 | 1.90 | 2.03 |
| 2.11 | 2.02 | 1.88 | 2.01 | 2.05 | 2.09 |
| 2.12 | 1.98 | 1.99 | 2.00 | 2.1 | 2.12 |

≥ 2.0   < 2.0 & ≥ 1.7   < 1.7

**OK**

**(B)** **BPC-NoCYCLO condition**

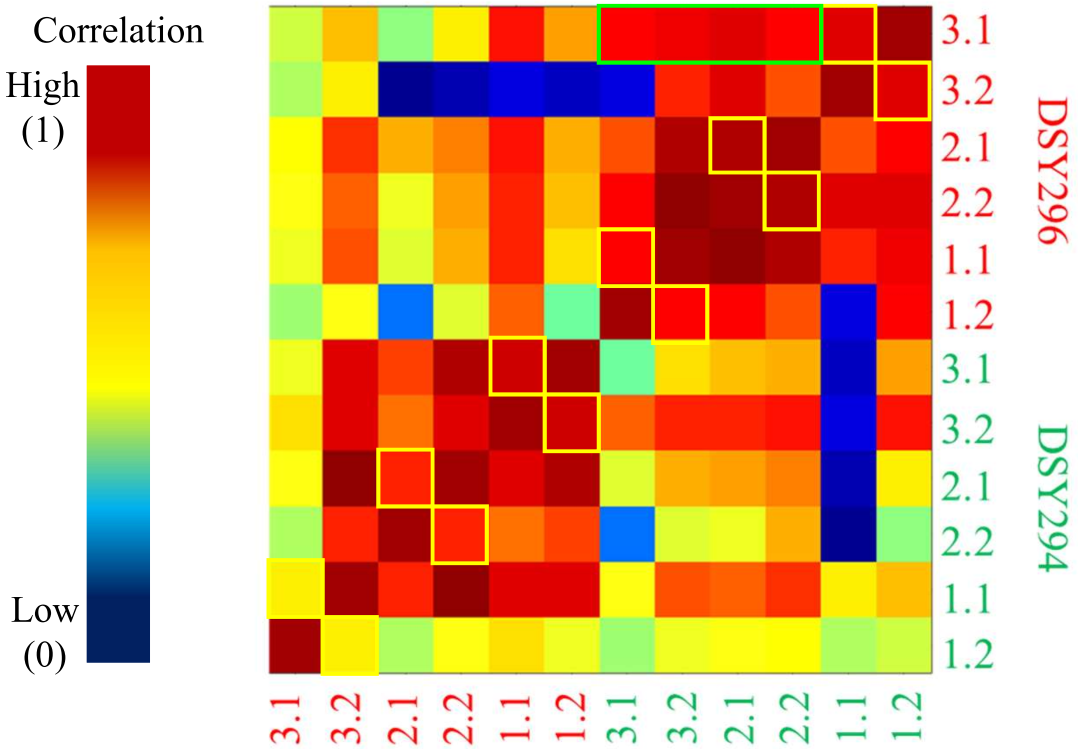


**Supplementary Figure 1: Quality controls.** A. First quality control QC1: Identification log(scores). Each spectrum is compared to all the reference spectra contained in the Bruker databases. For the fungal species, the log(score) must be equal or higher than 1.7 (yellow and green) to accept a proposed identification. Ba. Second quality control QC2: verification of the technical and biological variability. This panel represents an example of the second quality control for the pair of strain DSY294/DSY296. The spectra were acquired in the BPC-NoCyclo condition with the bead-based protocol, using 10µL of formic acid and 0.5mL of fungal suspension. The CCI matrix represents the similarities between the spectra. The red colors represent strong similarities whereas the blue colors represent weak similarities. The spectra from the biological replicates of the resistant strain of the couple, each in technical duplicate, are written in red. The spectra from the biological replicates of the susceptible strain of the couple, each in technical duplicate, are written in green. To be conserved, a spectrum must meet 2 conditions: 1. A spectrum must obtain a CCI score above 0.75 when compared to its technical duplicate (yellow box). 2. A spectrum must obtain a mean of CCI above 0.5 when compared to all its biological duplicates (green box).
