# Supplementary material for: Machine Learning Approach for Candida albicans Fluconazole Resistance Detection Using Matrix-Assisted Laser Desorption/Ionization Time-of-Flight Mass Spectrometry
Source: Front Microbiol. 2020 Jan 14;10:3000. doi: 10.3389/fmicb.2019.03000 (PMC6971193; doi:10.3389/fmicb.2019.03000)
Supplement: Supplementary file 3 [file Image_2.pdf]

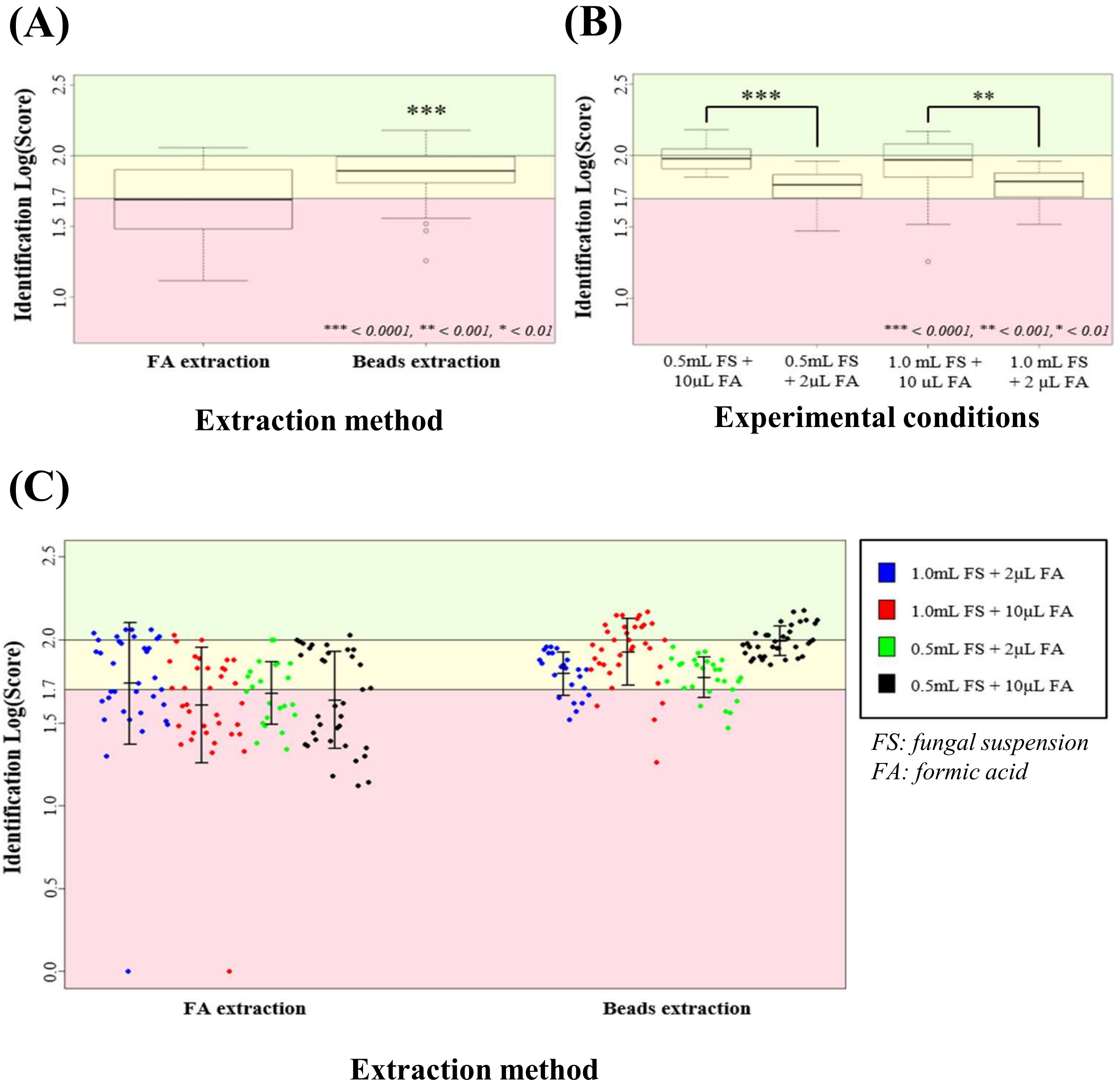

Supplementary Figure 2: Comparison of protein extraction protocols. The accuracy of the identification is expressed in log(scores). For a fungal species, the accuracy of the identification is sufficient if the log(score) is higher than or equal to 1.70 (yellow and green boxes), and excellent if higher than or equal to 2.0 (green boxes). Welch Two sample t-tests and Two-Way Crossed Analyses of variances (ANOVA) were performed, followed by an ad hoc Tukey HSD test were performed to compare the protocols' results. A. Comparison of the accuracy of identification obtained after a formic acid (FA) extraction and a glass beads-based extraction. B. Comparison of the impact of the FA volume and fungal solution (FS) volume in a glass beads-based extraction on the identification accuracy. C. Comparison of all the proteins extraction protocols tested. Each dot corresponds to a single replicate.
