# Supplementary material for: Machine Learning Approach for Candida albicans Fluconazole Resistance Detection Using Matrix-Assisted Laser Desorption/Ionization Time-of-Flight Mass Spectrometry
Source: Front Microbiol. 2020 Jan 14;10:3000. doi: 10.3389/fmicb.2019.03000 (PMC6971193; doi:10.3389/fmicb.2019.03000)
Supplement: Supplementary file 4 [file Table_1.pdf]

Supplementary Table 1 : MALDI-TOF MS settings used for the spectra acquisition

Parameters	Values
**Mode**	Linear positive
**Mass range detection**	2072 – 20000 m/z
**Laser frequency**	60 Hz
**Ion source 1**	20 kV
**Ion source 2**	18.10 kV
**Lens**	6 kV
**Laser shots**	600 shots in 100 shot steps
